# Leaf functional traits of *Parrotia subaequalis* from different environments in eastern China

**DOI:** 10.1002/pei3.70001

**Published:** 2024-08-24

**Authors:** Lifang Zhang, Mingjian Yu, Yanming Fang

**Affiliations:** ^1^ Zhejiang Academy of Forestry Hangzhou China; ^2^ College of Life Science Zhejiang University Hangzhou China; ^3^ Key Laboratory of Subtropical Forest Biodiversity Conservation, State Forestry Administration, Co‐Innovation Center for Sustainable Forestry in Southern China, College of Biology and the Environment Nanjing Forestry University Nanjing China

**Keywords:** bilateral symmetry, forest environmental factors, leaf functional traits, power law, rare and endangered plant

## Abstract

Functional traits are important in understanding how plants respond and adapt to their immediate environment. *Parrotia subaequalis* is a highly endangered arbor species found throughout eastern China, primarily inhabiting hillsides and valleys, yet, little is known about the variation in leaf traits across these environments. In the present study, we tested this by comparing leaf surface area, leaf weight, leaf length, leaf symmetry and leaf mass per unit area, as well as the relationship between leaf traits and environmental factors and the scaling relationship between leaf surface area versus leaf dry mass. We observed significant differences in leaf surface area, weight, and length among the population sites, and these variables were strongly affected by environmental factors, especially high mean annual temperatures in hillside habitats and high mean annual precipitation in valley habitats. The scaling exponents remained numerically variant among the 10 populations, with different slopes greater than 1.0, and the scaling exponents increased significantly with hillside habitats. These metrics correlated with soil thickness associated with different habitat types. The areal ratio (AR) values in all populations deviated from 1, indicating that the two lamina sides were asymmetrical. The standardized symmetry index (SI) values displayed significant variation, especially in leaves from hillside habitats with a high degree of asymmetry. Collectively, our findings demonstrated that leaf functional traits exhibit considerable variability in response to different environmental contexts and provide valuable reference data that could be useful for conserving this endangered species.

## Graphical abstract

Our findings demonstrate that leaf functional traits exhibit considerable variability in response to different environmental contexts and provide valuable reference data that could be useful for the conservation of this endangered species.

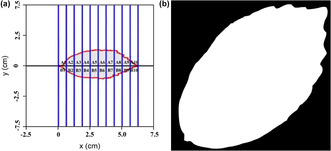

## 1 INTRODUCTION

Plant functional traits refer to adaptive and effective characteristics for the survival, growth, and reproduction of plants that can respond to changes in environmental factors and directly participate in ecosystem processes, thereby affecting ecosystem services (Balvanera et al., 2006; Eviner & Chapin, 2003; Liu & Ma, 2015; Violle et al., 2007). Plant functional traits are predictors of organism performance and represent adaptations to variations in the physical and biotic environments, (de Bello et al., 2010) and are central to plant‐environment interactions, as they enable population adaptation by regulating resource utilization (Hikosaka et al., 2014; Wang et al., 2018). Leaf traits determine many aspects of plant functional, including rates of photosynthesis and transpiration, investment costs for leaf construction, plant nutrient requirements, and resilience to temperature extremes, among others. Traits values vary both within and among species and along environmental gradients. Leaf traits such as leaf surface area, leaf weight, leaf dry mass per unit area (LMA), and leaf length are known to be common plastic traits. These traits reflect the main differences in the survival strategies adopted by plants in various environments to acquire resources (Wright et al., 2004; Zhang et al., 2016). For example, leaf surface area can affect leaf temperature via its effect on boundary‐layer conductance. Large leaves have a low rate of heat exchange with their surroundings, allowing more effective transpiration cooling in hot and wet climates, while potentially risking heat damage in dry climates or risking frost damage in cold climates (Gates, 1968). Leaf weight affects the ability of plants to intercept light and capture carbon (Parkhurst & Loucks, 1972). Leaf size affects its internal support tissue and chemical composition of the leaves and, ultimately, affects the physiological activity and leaf function of plants in different environments, resulting in better adaptation to the environment (Niinemets et al., 2006).

Additionally, LMA reflects different aspects of leaf carbon‐capture strategies. The leaf economics spectrum embodies the trade‐off between LMA and lifespan: many species growing in drought‐prone habitats with limited water or nutrient availability have high LMA, and their longer leaf lifespan reduces water absorption and nutrient uptake requirements (Bhusal et al., 2020). Leaves exposed to high levels of sunlight tend to exhibit higher LMA, whereas those exposed to shaded environments exhibit lower LMA (Blonder et al., 2011; Feldman et al., 2017; Osnas et al., 2018). In most vascular plants, the major components of LMA, leaf weight (*W*) and leaf surface area (*A*), are structural properties that affect important physiological processes such as photosynthesis, respiration, and transpiration (Calvo‐Alvarado et al., 2008). The relative changes in these two traits can be described as a “power law” taking the form: *W* = *αA* ^*β*^, where *α* is the normalization constant and *β* is the scaling exponent (Niklas et al., 2007; Niklas & Christianson, 2011). This formula indicates that the per‐unit investment of dry mass is size‐dependent on the amount of light‐capturing leaf surface (Pan et al., 2013; Sack et al., 2003). Several theoretical explanations for the numerical value of the scaling exponent between leaf area and dry mass have been proposed in recent studies. First, the scaling exponent of leaf area versus dry mass should be close to unity (i.e., *b* = 1.0), and this isometric scaling relationship should be insensitive to environmental differences (Sun et al., 2006). Second, according to the “diminishing returns” hypothesis, the scaling exponent for leaf area versus dry mass is predicted to be less than unity (i.e., *b* < 1.0), indicating that gains in leaf area do not keep pace with increasing leaf mass investment (Milla & Reich, 2007). The third hypothesis is that the scaling exponent of leaf area versus dry mass should increase disproportionately with increasing leaf dry mass (i.e., *b* < 1.0).

Moreover, leaf shape is also considered as an important functional trait affected by environmental factors. For example, in a common garden experiment with different California white oak (*Quercus lobata*) provenances, Ramirez et al. (2020) found that leaf shape variation had functional implications and was correlated with light‐related factors. In oak plants, the variation in leaf dissection and specific leaf area could be explained by adaptation. And, the variation in leaf dissection is associated with photosynthetic rate, leaf shape has a positive relationship between leaf dissection and photosynthetic rates. Thus the variation in leaf shape have functional consequences and influence how valley oaks cope with environmental stress. Leaf shape has been linked to the photosynthetic rates and temperature optima in some plants. Many plants have leaves that exhibit bilateral symmetry. Thompson (1917) studied the leaf shape of *Begonia daedalea* Lem., and hypothesized that the left side of the lamina is proportional to its right side. Therefore, both leaf trait and bilateral symmetry are often considered when conducting leaf functional trait studies. Early work focused primarily on the traditional morphology. However, this approach lacks an accurate graphical representation of shape variation and may overlook important mechanisms by which leaf traits adapt to environmental changes. We used a method to automatically extract the planar coordinates of an object's edge using an M file in Matlab to directly measure the extent of bilateral symmetry (Shi, Ratkowsky, et al., 2018; Shi, Zheng, et al., 2018).

Nevertheless, it is noteworthy that the majority of functional traits studies have focused on whole ecosystem function, plant communities, and economic crops, however, litter is known about endangered species, particularly plants such as *Parrotia subaequalis, which* grow at different elevations and habitats in subtropical forests. *Parrotia subaequalis* is a rare and endangered tree endemic to China. This deciduous species of the family of Hamamelidaceae (Chang, 1997; Hao & Wei, 1998) is an important living fossil of an angiosperm lineage that first evolved 67 million years ago. Within China, it has been described as a “plant species with extremely small populations” (Ellie et al., 2016; Wang & Xie, 2004). *P. subaequalis* occurs in eastern China, where it exhibits a disjunct distribution in the Anhui, Jiangsu, Henan, and Zhejiang Provinces (Table 1), and restrict to a narrow range of ecosystems that are characterized by high heterogeneity (e.g., elevation, temperature, and moisture). In these areas, *P. subaequalis* often struggles to compete with other tree species and is consequently confined to less favorable environments (Gong et al., 2012; Ren et al., 2012; Zhang et al., 2016).

**TABLE 1:** Population locations, sample sizes, habitats, and dates from which leaves from *Parrotia subaequalis* were collected.

No.	Population code	Sample location	Altitude (m)	Latitude (°N)	Longitude (°E)	Habitat	Sample size	Site slope (°)	Mean annual precipitation (mm)	Mean annual air temperature (°C)	Air humidity (%)	Soil thickness (cm)	Soil water availability (%)
1	AJ	Anji, Zhejiang	820	30°23′	119°24′	Hillside	150	58	1220.1	15	55	16.7	0.14
2	XY	Xinyang, Henan	192	31°27′	115°16′	Hillside	150	60	1171.8	15.8	46	14.1	0.12
3	YXI	Yuexi (Huangwei), Anhui	449	31°06′	116°19′	Hillside	150	55	1290.4	14.6	58	20.9	0.22
4	CH	Changhua, Zhejiang	864	30°10′	119°11′	Hillside	150	62	1123.6	14	65	16.3	0.15
5	JD	Jingde, Anhui	653	30°25′	118°35′	Hillside	150	75	1286.1	14.1	60	25.6	0.12
6	YX	Yixing, Jiangsu	252	31°14′	119°44′	Hillside	150	65	1294.6	14.1	50	26.1	0.16
7	SC	Shucheng, Anhui	584	31°4′	116°33′	Valley	150	3	1380.5	12.8	70	10	0.24
8	TC	Tongcheng, Anhui	270	31°05′	116°51′	Valley	150	6	1419.9	12.2	68	24	0.27
9	JZ	Jinzhai, Anhui	450	31°12′	115°54′	Hillside	150	60	1290.5	15.9	62	24.3	0.18
10	YXII	Yuexi (Hetu), Anhui	313	30°49′	116°02′	Hillside	150	35	1290.4	14.6	52	17.6	0.17

In present study, we collected leaves from 10 populations spanning a range of environmental conditions and used geometric morphometry methods to assess variations in leaf functional traits (leaf area, weight, length, leaf mass per area, and leaf shape) to better understand how leaf functional traits vary in response to environmental conditions in *P. subaequalis* populations. With these data, we hypothesized that (1) leaf functional traits will follow the adaption of hillside and valley habitats and display more interspecific variances, and populations in valley habitats will have larger leaf trait values, (2) environmental factors, particularly mean annual precipitation (MAP), mean annual temperature (MAT), air humidity and altitude will have significant, but different effects on leaf traits, and (3) the scaling exponent will differ among different types of habitats.

## 2 MATERIALS AND METHODS

### 2.1 Population sampling

We collected *P. subaequalis* leaf samples from Anhui (six populations), Jiangsu (one population), Henan (one population), and Zhejiang (two populations) provinces in China. For each population, we collected 10 individuals and sampled 15 leaves from each individual. Leaf sampling occurred in July, when the growth of *P. subaequalis* trees had already begun. Trees at least 50 m apart were sampled to avoid sampling closely related individuals within a population. Individual tree characteristics (sampling height, tree age, and diameter at breast high) are represented in Table S1. We collected leaves from the crown in four cardinal directions and the four outermost branches, both from sunny and shady locations within the canopy in each population. Population locations, sample sizes, habitat types, and environmental factors are listed in Table 1. Leaf images from the 10 populations of *P. subaequalis* are shown in Figure S1.

### 2.2 Leaf functional traits data

We measured the leaf surface area, length, weight, and leaf shape of 10 populations located in the four Chinese provinces assessed in this study. Leaf surface area and length were measured for mature leaves using non‐destructive methods. Specifically, we traced the leaf outline onto paper and later digitized these tracings. Leaf weight was measured as dry weight using SQP (QUINTIX Equipment Limited Company, Beijing, China; *d* = 0.0001 g). The LMA was calculated by dividing the leaf weight by the leaf surface area.

### 2.3 Climate data

We selected our study sites to cover a wide geographic range. The study sites spanned an area that stretches from Mount Tianmu to Mount Dabie. Moreover, the study sites encompassed a wide variety of environmental conditions. We recorded the data for seven environmental variables, including site‐specific and soil‐related environmental factors, for each population. The altitude of our study sites ranged from 192 to 864 m a.s.l. The slopes of the sites ranged from 3° to 75°. The mean annual precipitation (MAP) ranged from 1171.8 to 1419.9 mm. Air humidity ranged from 46% to 70% and mean annual air temperature (MAT) reached a maximum of 15.9°C and a minimum of 12.2°C in 2020 (Data from the China Meteorological Data Sharing Service System (CMDSSS); www.Cdc.cma.gov.cn) (Li & Zhang, 2015). Meanwhile, we used the method of pin insertion to measured soil thickness and based on the results, the soil thickness of the 10 population sites ranged from 10 to 26.1 cm (Li et al., 2020). We measured the characteristic curve of soil moisture to analyze soil water availability. Soil water availability ranged from 0.12% to 0.27% among the 10 population sites (Li et al., 2006).

### 2.4 Calculations and statistics

We assumed that leaf weight (*W*, g) and leaf surface area (*A*, cm^2^) were related as defined by the following power function (Milla & Reich, 2007):

(1) $$W=\alpha \;A^{\beta }.$$

To facilitate computation and visualization, we log‐linearized the above function. In doing so, we converted the power exponents to linear slope scores, wherein the slope (*b*) determined the relationship between leaf weight and leaf area:

(2) $$\ln\;\left(W\right)=a+b\;\ln\;\left(A\right).$$

With this formulation of the original power function, we would expect a positive slope (*b*) if the LMA was positively associated with leaf area:

(3) $$\frac{W}{A}=\alpha A^{b-1}.$$

After log‐transforming the data for *A* and *W*, the scaling relationship was determined using the reduced major axis (RMA). Unlike ordinary linear regression, this alternative linear‐function fitting method estimates the slope by taking the square root of the ratio of the variance of the response variables to that of the independent variables (Hui et al., 2010). Using RMA regression, we determined the scaling exponent (slope, *b*) and constant (*a*) of the log–log‐linear function. We used bootstrap percentile methods to calculate the 95% confidence interval of the slope (*b*) and test its significance level (Davison & Kuonen, 2003; Efron & Tibshirani, 1993). Significance was determined using an *α* = 0.05 threshold, indicating the level of significance in determining if the slope differed from 1. The leaf traits (leaf area, leaf weight, and leaf length) of the 10 populations, totaling 1500 leaves, were tested for normality, and a one‐way analysis of variance (ANOVA) was used to test for significant differences. The multiple analysis of significance (LSD) method and Bonferroni correction were used to compare differences between the leaf traits of the 10 populations.

**FIGURE 1:**
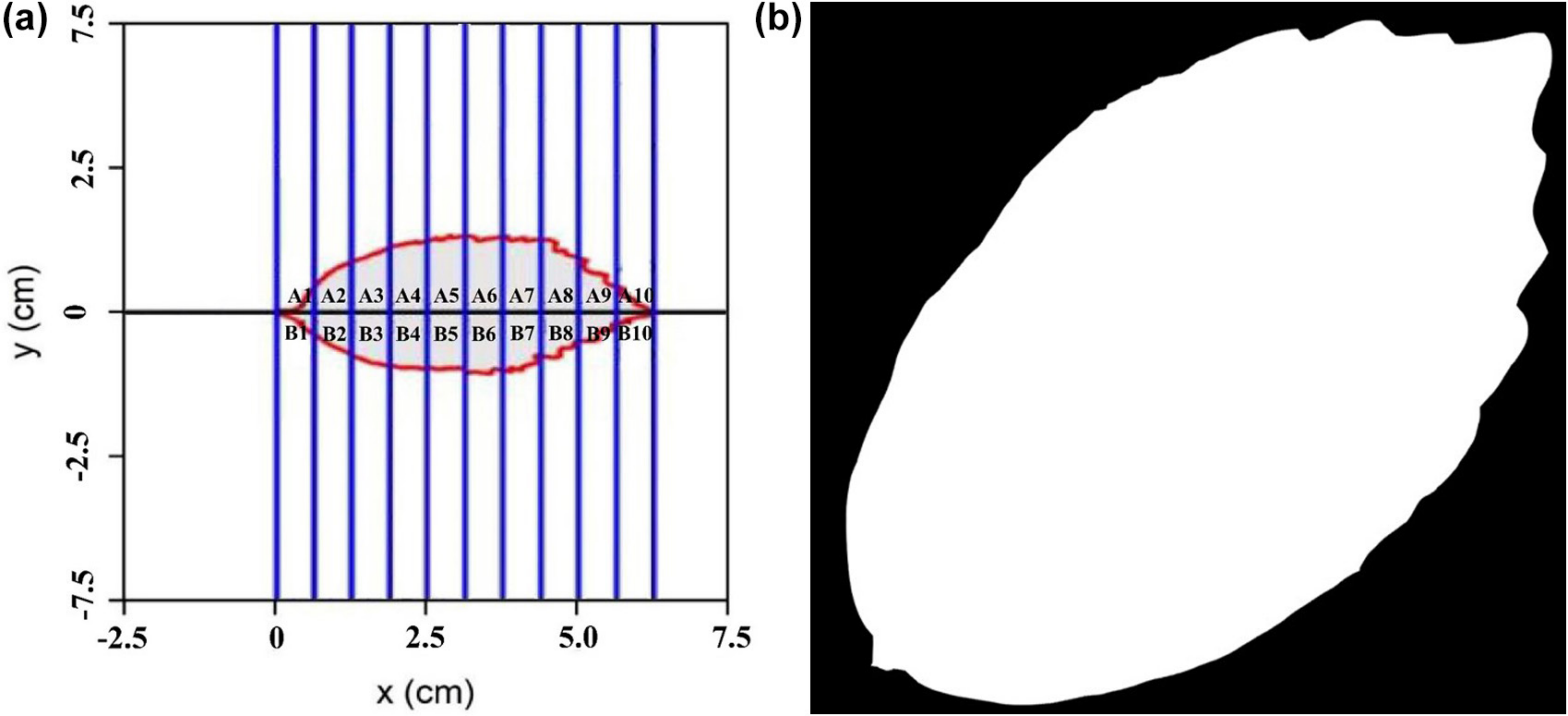
Illustration of the calculation for the areal ratio (AR) of the left side to the right side, and the standardized index (SI) for measuring leaf bilateral symmetry. A1 to A10 represent the areas of the upper (left) subregions (the intersections between strips that were formed by the adjacent blue vertical lines with the left side of this leaf), and B1 to B10 represent the areas of the lower (right) subregions. AR = (A1 + A2 + A3 + A4 + A5 + A6 + A7 + A8 + A9 + A10)/(B1 + B2 + B3 + B4 + B5 + B6 + B7 + B8 + B9 + B10) and, SI = [|A1 − B1|/(A1 + B1) + |A2 − B2|/(A2 + B2) + |A3 − B3|/(A3 + B3) + |A4 − B4|/(A4 + B4) + |A5 − B5|/(A5 + B5) + |A6 − B6|/(A6 + B6) + |A7 − B7|/(A7 + B7) + |A8 − B8|/(A8 + B8) + |A9 − B9|/(A9 + B9) + |A10 − B10|/(A10 + B10)/10]. In this figure, we used 10 strips to conveniently showing the calculation process, and during the actual calculation we used 1000 strips within a given leaf during the actual calculation, which better reflects the information regarding bilateral symmetry (a). Representative picture of processing formats of the *P. subaequalis* populations (b).

Because of its practicality and suitability, we followed the approach of the simplified Gielis equation to calculate the bilateral symmetry (BA) indicators, namely the standardized index (SI) and the areal ration (AR). Each leaf was divided into two equal parts (upper and lower sides or left and right sides) from the apex to the base. Subsequently, uniform strips were obtained by digitally cutting the leaves (Figure 1; Figure S2). Here, let *n* denoted the number of strips, and let *A*_*i*_ and *B*_*i*_ represented the upper and lower intersection areas, respectively, of the *i*‐th strip within the leaf. The extent of bilateral symmetry was estimated by calculating the variation in the standard deviation, the root mean squared error (RMSE), and a standardized symmetry index (SI) using the following two equations, respectively:

(4) $$\text{RMSE}=\sqrt{\sum_{i=1}^{n}{\left(A_{i}-B_{i}\right)}^{2}}/n.$$

(5) $$\mathrm{SI}=\frac{1}{n}\sum_{i=1}^{n}\frac{|A_{i}-B_{i}|}{A_{i}+B_{i}}.$$

In addition, we used a second index, the areal ratio (AR), to measure bilateral symmetry, where *A* represents the area of the upper (left) side of a leaf, and *B* referred to the area of the lower (right) side of the leaf:

(6) AR=A/B.

We used the R function “bilat. Measure” to calculate the RMSE, SI, leaf area, and AR from the left side to the right side. ANOVA and the multiple analysis of significance (LSD) method were used to the analyze of leaf functional traits in different sample populations. Linear regression analysis (LM) was used to test the correlations between leaf surface area, length, weight, scaling exponents, and environmental factors. All data were analyzed and graphically completed using R software (version 3.5.0) (R Development Core Team, 2020).

## 3 RESULTS

### 3.1 Leaf functional traits

Overall, the leaf traits of the 10 populations showed high intraspecific variation (5.50–27.00‐fold) (Table S2). We observed an average leaf surface area of 11.78 ± 5.60 cm^2^, an average leaf length of 5.36 ± 1.31 cm, and an average leaf weight of 0.07 ± 0.04 g. ANOVA revealed significant leaf traits' intraspecific differences in leaf surface area (Figure S3a), leaf length and leaf weight (Table S3; Figure S3b,c) among populations. The leaf surface area of each population was as follows, TC population (14.63 ± 7.53 cm^2^) that grew in the valley habitat had a higher value than the other populations. Similarly, individual population leaf length varied significantly across populations, with the mean lengths of the TC (6.32 ± 1.57 cm) and SC population (5.15 ± 1.24 cm) populations having greater leaf length than other populations such as the YXII (5.03 ± 1.24 cm) and JD (4.75 ± 1.11 cm) populations. This indicated that the leaf length displayed a declining trend from valley to hillside habitats. Leaf weight also varied by population, with TC, SC, JZ, and JD populations exhibiting significantly lower weights than the other populations.

**FIGURE 2:**
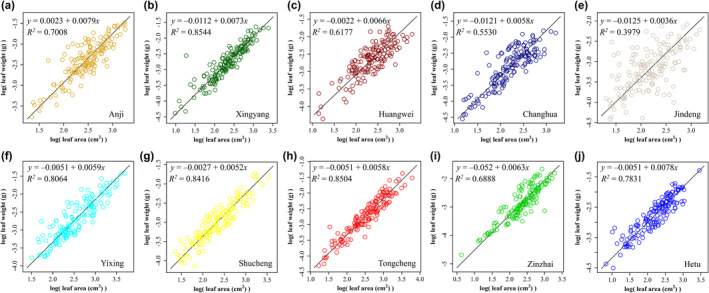
The linear fit between the logarithm of leaf weight and leaf area for the populations from (a) Anji; (b) Xinyang; (c) Huangwei; (d) Changhua; (e) Jingde; and (f) Yixing; (g) Shucheng; (h) Tongcheng; (i) Jinzhai; and (j) Hetu (see Table 1 for details). In each panel, *y* represents the natural logarithm of leaf weight in g; *x* represents the natural logarithm of leaf area in cm^2^; *R* ^2^ represents the degree of fit for the regression model.

Our regression analysis revealed a positive relationship between leaf surface area and leaf weight across all 10 populations (Figure 2). Of the 10 populations assessed, the coefficients of fitting degree (*r* ^2^) were greater than 0.70 for six populations. However, we observed significant variations in the LMA across populations (Figure S3d). The order of LMA, from highest to lowest, was as follows: AJ population > YXII population > CH population > XY population > JZ population > YXI population > YX population > SC population > TC population > JD population. In the hillside habitats, the AJ, YXII, CH, and XY populations did not show significant differences, however, compared to that of the valley habitats, the TC and SC populations exhibited high levels of significance.

### 3.2 Leaf functional traits with environmental conditions

**FIGURE 3:**
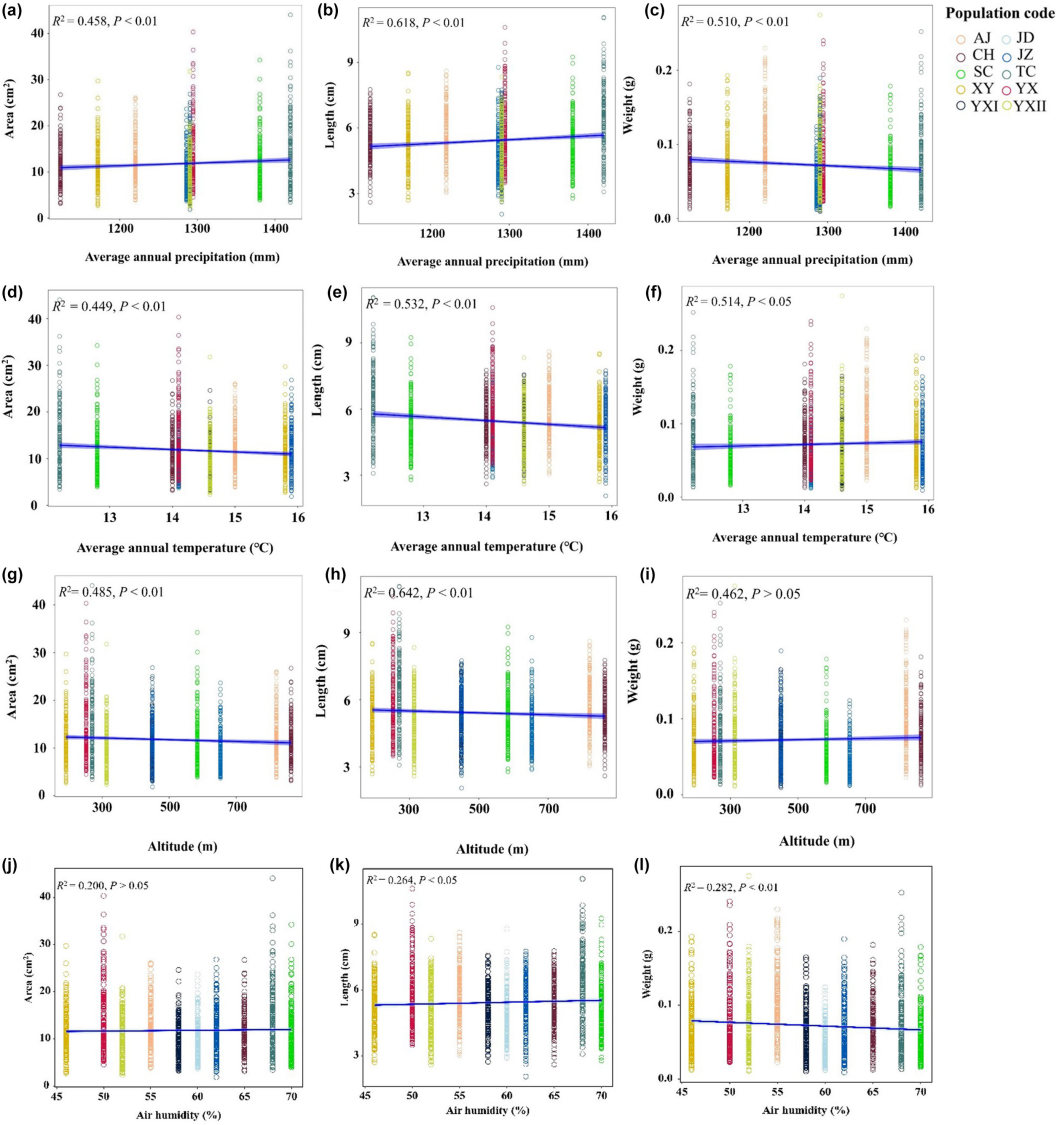
Correlation analysis between leaf area, leaf weight, and leaf length and average annual temperature, average annual precipitation, altitude, and air humidity. (a) Correlation analysis between leaf area (cm^2^) and average annual precipitation (mm). (b) Correlation analysis between leaf length (cm) and average annual precipitation (mm). (c) Correlation analysis between leaf weight (g) and average annual precipitation (mm). (d) Correlation analysis between leaf area (cm^2^) and average annual temperature (°C). (e) Correlation analysis between leaf length (cm) and average annual temperature (°C). (f) Correlation analysis between leaf weight (g) and average annual temperature (°C). (g) Correlation analysis between leaf area (cm^2^) and altitude (m). (h) Correlation analysis between leaf length (cm) and altitude (m). (i) Correlation analysis between leaf weight (g) and altitude (m). (j) Correlation analysis between leaf area (cm^2^) and air humidity (%). (k) Correlation analysis between leaf length (cm) and air humidity (%). (l) Correlation analysis between leaf weight (g) and air humidity (%). Different colored continuity dots represent the sampled populations of *P. subaequalis* in China.

**FIGURE 4:**
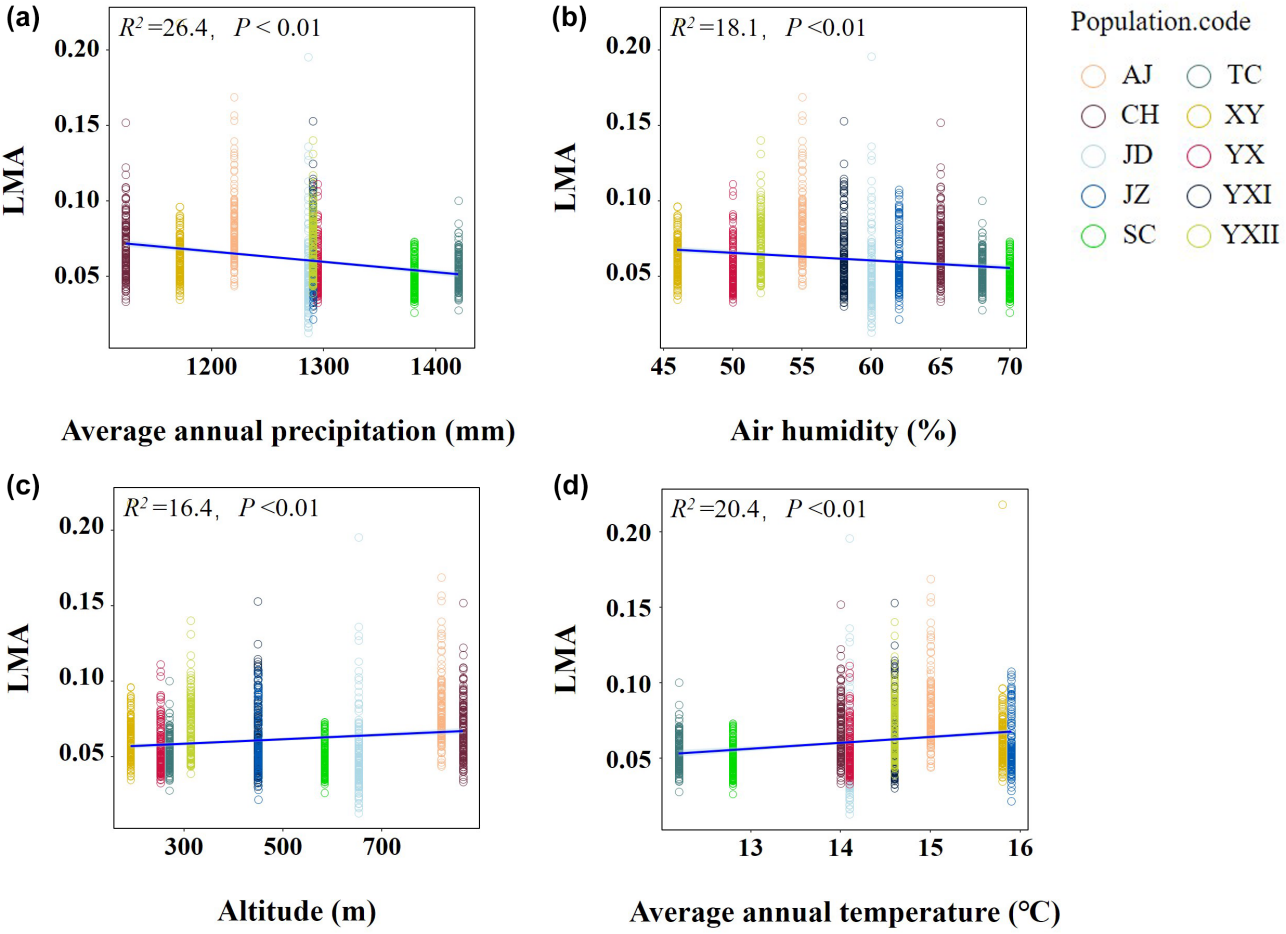
Correlation analysis between leaf dry mass per unit area (LMA) and average annual precipitation (a), air humidity (b), altitude (c) and average annual temperature (d). Different colored continuity dots represent the sampled populations of *P. subaequalis* in China.

Comparing the correlations between environmental factors and leaf traits, we founded that the leaf surface area of the 10 populations was positively correlated with MAP, soil thickness, air humidity, and soil water availability (Figure 3a,j; Figure S4d,g), while negatively correlated with MAT, altitude, and site slope (Figure 3d,g; Figure S4a) (*p* < .01). Leaf weight was negatively correlated with MAP, air humidity, soil thickness, soil water availability, and site slope (Figure 3b,k; Figure S4c,f,i) (*p* < .01, *p* > .05), and positively correlated with MAT, altitude (Figure 3e,h) (*p* < .05, *p* > .05). Leaf length was positively influenced by MAP, air humidity, soil thickness, and soil water availability (Figure 3c,l; Figure S4e,h) (*p* < .01), and negatively affected by MAP, site slope and altitude (Figure 3f,i; Figure S4b) (*p* < .01). Meanwhile, LMA was negatively correlated with MAP, air humidity, soil water availability, and soil thickness (Figure 4a,b; Figure S5b,c) (*p* < .01), and positively affected by MAT, altitude, and site slope (Figure 4c,d; Figure S5a) (*p* < .01).

The MAP, air humidity, and soil water availability values for the SC and TC populations were higher than those for the other sites, and these two populations had higher leaf surface area and leaf length, but not a higher leaf weight. The MAT values and site slopes for the SC and TC populations were lower than those for the other sites and they had a higher leaf surface area and leaf length but not a higher leaf weight. Thus, populations growing in a valley habitats have a higher leaf surface area, leaf length, and lower leaf weight. However, the altitude and soil thickness values for the SC and TC populations were lower or higher than those at the other sites, respectively, with higher or lower leaf surface area, leaf length, and leaf weight.

### 3.3 Scaling exponent with environmental conditions

**FIGURE 5:**
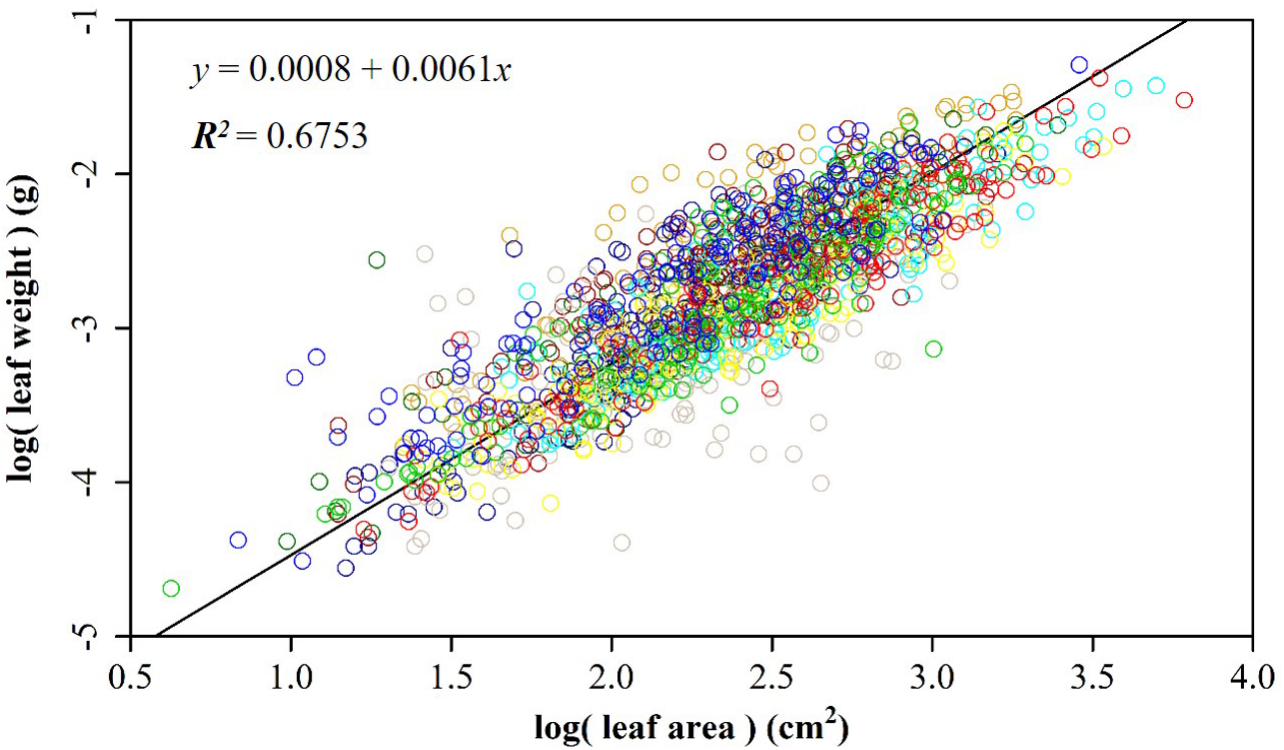
The linear fit between the natural logarithm of leaf weight and area for an analysis encompassing 10 populations of *Parrotia subaeualis*. Each population is visually distinguished using distinct colors.

**FIGURE 6:**
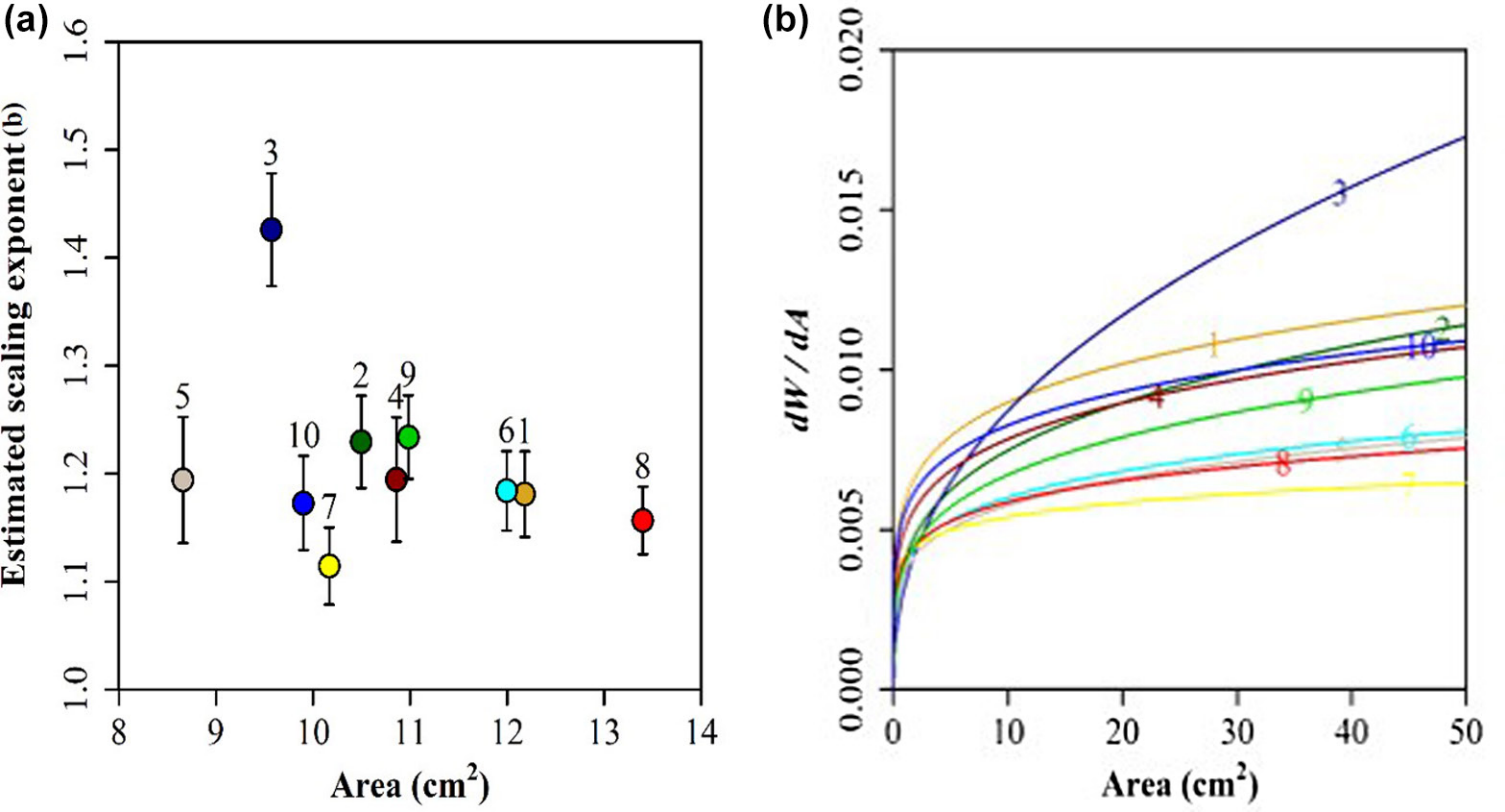
Mean slopes (*b*) ± SD of the linearized power function *W* = *aA* ^*β*^, which is associated with the mean leaf area across 10 populations (a) and the relationship between the change in leaf weight and the change in leaf surface area for each of the 10 populations of *Parrotia subaequalis*. Reflection on the scenarios depicted to the comparison of reciprocal *W* to *A* (b). The number 1 represents the AJ population and so on.

After pooling the 1500 data points collected across the 10 populations, we obtained a linearized equation of ln (*W*) = 0.0008 + 0.0061 ln(*A*), with an *R* ^2^ = .6753 (Figure 5). Leaf area and leaf weight demonstrated positive relationships with each other. Among the 10 populations, leaf area (*A*) ranged from 1.87 in the JZ population to 44.04 cm^2^ in the TC population. This range spanned two orders of magnitude, highlighting the considerable variation in leaf area among the studied populations. The exponent *b* for the power function $\frac{W}{A}=\alpha A^{b-1}$ averaged 1.21 for the 10 populations, as determined through 3000 bootstrap replications (Table 2). However, *b* varied among the populations, ranging from 1.11 to 1.43 (with a 95% confidence interval (CI) of 1.04–1.53). Notably, the YXI population had a significantly higher slope than all other populations (i.e., *b =* 1.42, 95% CI = 1.32–1.52), while the SC and TC populations had a significantly lower slopes than certain populations (i.e., *b =* 1.11, 95% CI = 1.04–1.18) (Table 3; Figure 6a). In addition, we calculated the slopes of the reciprocals of leaf weight and leaf surface area for the 10 populations based on the aforementioned analysis (Figure 6b). Importantly, the YXI and SC populations exhibited slopes different from those of the other populations, suggesting the unique characteristics or dynamics of these specific populations. Moreover, scaling exponent *b* showed positively correlations with MAT, site slope, soil water availability (Figure S6b, e.g., *p* > .05) and soil thickness (Figure S6d, *p <* .05), and negatively correlations with MAP, air humidity, and altitude (Figure S6a,c,f, *p* > .05).

**TABLE 2:** Estimates of the *a* and *b* parameters for each population of *Parrotia subequalis*.

	Population code	Estimate	Mean	SD	Median	Lower CI	Upper CI
*a*	AJ	−5.29	−5.29	0.10	−5.29	−5.51	−5.10
*a*	XY	−5.64	−5.64	0.11	−5.64	−5.86	−5.43
*a*	YXI	−6.06	−6.07	0.10	−6.07	−6.28	−5.86
*a*	CH	−5.47	−5.47	0.14	−5.47	−5.75	−5.20
*a*	JD	−5.78	−5.78	0.13	−5.77	−6.04	−5.54
*a*	YX	−5.70	−5.70	0.09	−5.71	−5.91	−5.52
*a*	SC	−5.59	−5.59	0.08	−5.59	−5.76	−5.43
*a*	TC	−5.64	−5.64	0.08	−5.64	−5.80	−5.49
*a*	JZ	−5.75	−5.75	0.09	−5.75	−5.95	−5.59
*a*	YXII	−5.35	−5.35	0.10	−5.36	−5.56	−5.15
*b*	AJ	1.18	1.18	0.04	1.18	1.10	1.26
*b*	XY	1.23	1.23	0.04	1.23	1.14	1.31
*b*	YXI	1.43	1.43	0.05	1.43	1.33	1.53
*b*	CH	1.19	1.19	0.06	1.19	1.08	1.31
*b*	JD	1.19	1.19	0.06	1.19	1.09	1.31
*b*	YX	1.18	1.18	0.04	1.18	1.11	1.26
*b*	SC	1.11	1.11	0.04	1.12	1.04	1.19
*b*	TC	1.16	1.16	0.03	1.16	1.09	1.21
*b*	JZ	1.23	1.23	0.04	1.23	1.17	1.32
*b*	YXII	1.17	1.17	0.04	1.17	1.09	1.26

**TABLE 3:** The significance of exponent *b* relative to unity (1.0): “+” = significantly greater than 1; “−” = significantly less than 1; “ns” = not significantly different from 1.

Population code.	V1	V2	V3	V4	V5	V6	V7	V8	V9	V10
AJ	ns	ns	−	ns	ns	ns	ns	ns	ns	ns
XY	ns	ns	−	ns	ns	ns	+	ns	ns	ns
YXI	+	+	ns	+	+	+	+	+	+	+
CH	ns	ns	−	ns	ns	ns	ns	ns	ns	ns
JD	ns	ns	−	ns	ns	ns	ns	ns	ns	ns
YX	ns	ns	−	ns	ns	ns	ns	ns	ns	ns
SC	ns	−	−	ns	ns	ns	ns	ns	−	ns
TC	ns	ns	−	ns	ns	ns	ns	ns	ns	ns
JZ	ns	ns	−	ns	ns	ns	+	ns	ns	ns
YXII	ns	ns	−	ns	ns	ns	ns	ns	ns	ns

*Note*: V1 to V10 represent the population codes. V1 represents the AJ population and so on.

### 3.4 Leaf shape symmetry

The bilateral symmetry of the leaf shape was assessed using the AR and SI values in most species. Our analysis revealed that the mean and median values for leaf symmetry values varied across populations. Specifically, in terms of the aspect ratio (AR), the median values for the XY, YXI, JD, TC, and YXII populations were significantly lower than 1, indicating a greater extent of deviation from a symmetrical shape compared with the other populations (Figure S3e). Our data indicated that the populations exhibited differences in the leaf surface area between the left and right sides, resulting in the degree of asymmetry varied significantly across populations (Figure S3f). This finding highlighted the fact that asymmetry is common among leaves across a range of environmental conditions. In some populations, such as the TC population, the AR was less than 1, indicating a greater deviation from a symmetrical shape. However, in other populations, such as the SC population, we observed a value greater than one, indicating a higher degree of symmetry.

## 4 DISCUSSION

### 4.1 Intraspecific variance of leaf traits

We hypothesized that leaf traits would vary among populations and environmental conditions and that populations in valley habitats would have larger leaf trait values. Indeed, the leaf traits (i.e., leaf surface area, leaf length, and leaf weight) of the 10 populations used in the present study differed significantly between sites with distinct environmental contexts, and populations had large values of leaf traits in valley habitats. In valleys, the leaves of the TC and SC populations were longer and had higher surface areas, but weighed less than those from plants growing in hillside habitats. These results suggested that leaves growing in valleys, which are often characterized by ample water availability, tend to have large leaf surface areas that can facilitate photosynthesis and increase the area available for gas exchange with the surrounding environment. In contrast, the leaves associated with the hillsides were smaller, thicker and had smoother leaf edges. Similar findings have been reported in an analysis of five bamboo species belonging to three functional groups (Sun et al., 2017).

We evaluated the relationship between leaf surface area and leaf weight to further explore this hypothesis. We observed a positive correlation between leaf surface area and leaf weight in all populations but with a relatively low *r* ^2^ value. In addition, the values of LMA also differed significantly among populations and environmental conditions. For example, the SC and TC populations in valley habitats exhibited the lowest LMA values. Conversely, the AJ, CH, and YXII populations in the hillside habitats exhibited the highest values. Variations in LMA were positively correlated with structural features that increased CO_2_ diffusion within the palisade mesophyll, indicating that higher LMA promoted a higher photosynthetic rate (Jurik, 1986; Lin et al., 2018; Osnas et al., 2018; Wu et al., 2018). These results aligned with the findings of Wright and Reich (2002) and Wang et al. (2021), who observed that species in dry and infertile soils also exhibited higher LMA, leaves with larger cell sizes, greater allocation of major veins, more layers of mesophyll cells, and higher cell mass densities (Flores et al., 2014; John et al., 2017). Higher LMA enables species to exhibit higher survival rates in resource‐poor environments.

### 4.2 Correlation between leaf traits and environmental factors

Our hypotheses led us to explore the environmental factors that lead to the variance in leaf traits. We found that the variables of leaf surface area and leaf length were positively and linearly related to MAP and air humidity, but negatively related to MAT and altitude. The variable of leaf weight was positively correlated with MAT and altitude, but negatively correlated with MAP and air humidity. These results were consistent with those of other studies. Dong et al. (2020) demonstrated that leaf surface area and leaf length were significantly and positively correlated with MAP, with an increase in MAP and air humidity, the leaf surface and length increased continuously. Moreover, LMA was negatively correlated with MAP and air humidity, and positively correlated with MAT and altitude. Plants in drought areas have decreased leaf size, which increases leaf production (Casper et al., 2001; Rosas et al., 2019). Additionally, leaf area and length showed a decreasing trend along the altitudinal gradient and MAT, whereas, leaf weight showed an increasing trend (Zhong et al., 2022). However, in other studies, leaf surface area increased with mean growing‐season temperature (mGDD0) and decreased with vapor pressure deficit (mVPD0) and soil pH (Dong et al., 2024).

In conclusion, the ecological adaptation strategies of *P. subaequalis* populations differ between different habitats. A series of morphological features are adopted in the hillside and valley habitats with high or low environmental factors, mainly including increasing leaf weight to improve mechanical resistance and reducing leaf surface area to reduce leaf surface damage caused by light energy capture. Low‐altitude plants rapidly obtained resources by increasing the leaf surface area and leaf length and reducing leaf weight.

### 4.3 Correlation between scaling exponent and environmental factors

The relationship between mass and area scaling has important implications for understanding the ability of plants to acclimate to environmental conditions (Pan et al., 2013). We hypothesized that the scaling exponent would be following the “diminishing returns” hypothesis and be insensitive to environmental differences. We found a range of variation in *M* to *A* scaling exponents among populations in this study. Indeed, the scaling exponents for leaf surface area to leaf mass were all substantially bigger than 1.0 across populations, thus providing evidence against these hypotheses. Environmental control of specific leaf areas induces variation in leaf allometry. Leaves are subjected to strong selective pressures in response to aridity, solar radiation, and nutrient availability, which affect their size and shape. The scaling exponents are expected to vary across environments as they balance the need for net carbon acquisition and protection against desiccation (Price & Enquist, 2007; Tomlinson et al., 2013).

Furthermore, according to the power function, LMA positively correlated with leaf surface area, implying a relationship between leaf surface area and the photosynthetic rate. However, the YXI and SC populations were significantly different from the other populations. The scaling exponent *b* values of populations YXI and SC showed the greatest difference, possibly reflecting the different habitat types observed at these sites. The *b* values showed that the XY, YXI, and AJ populations had higher slopes than the SC and TC populations, which grew in the valleys. The results of comparing the scaling exponent *b* showed that more species had a statistically significant decrease in LMA as leaf size increased, and large leaves had less surface area per unit dry mass than small leaves (Milla & Reich, 2007). *W* scales “faster” than *A*, and leaves in valley habitats show lower LMA than those growing in hillside habitats. Plant performances in valley habitats is limited by low temperatures, high irradiance, and moisture. Low temperatures limit transportation efficiency and thus, leaves may require a high investment in the transportation structure. Therefore, large leaves tend to have a larger fractional biomass investment in support structures than small leaves. On the other hand, small leaves produce smaller wind‐induced drag forces and have lower support needs, and thus a higher fraction of productive tissue. Additionally, a more gradual increase in the scaling exponent *b* values was related to MAP, site slope, soil thickness, and soil water availability, but, with low *r* ^2^ and *p* values. Thus, it is reasonable to infer that variations in the numerical values of the scaling exponent *b* were influenced by environmental factors.

### 4.4 Variances of leaf symmetry

Bilateral symmetry indicators, such as AR and SI can be effective measures for assessing the adaptability of leaves to their environment. The left and the right sides of an organism with bilateral symmetry (or of a bilaterally symmetry) are separate copies of a morphological structure that develops under the control of the same genome and under the same environmental conditions. Greater fluctuations in leaf shape may indicate a heightened level of adaptation to environmental conditions (Cuevas‐Reyes et al., 2018; Shi et al., 2020; Tucić et al., 2017). Shi et al. (2020) found that the AR values of vine species were not significantly different, however, the SI values had significant differences. These data showed that vines tend to generate a similar number of left‐and‐right‐skewed leaves, which may contribute to optimizing light interception. The present study provides evidence against the findings of these studies, because the AR values were approximately equal to 1.0. The leaves of most populations are typically not entirely bilaterally symmetrical and thus manifest larger SI values. Most *P. subaequalis* populations exhibited different degrees of bilateral symmetry. With the exception of the TC population, the SI values of the leaves across the 10 populations of *P. subaequalis* displayed significant variation, suggesting a lack of regular distribution of leaves across habitats, with plants from hillside habitats exhibiting a higher degree of asymmetry. This asymmetry may have resulted from the irregular distributions of leaves in response to environmental conditions such as elevation. According to a recent study, environmental conditions can alter leaf shape (Yu et al., 2019). Thus, differences in leaf shape among populations indicated that leaves with different shaped from different habitats might reflect different adaptive strategies for light competition.

## 5 CONCLUSION

By analyzing 1500 measurements obtained from 10 populations of *P. subaequalis*, we found that there were significant differences in leaf surface area, weight, and length among the different populations. Populations have large values in valley habitats. The variable of leaf surface area, weight, and length were also correlated with environmental factors, especially mean annual precipitation and mean annual temperature. We observed that the scaling relationship between leaf surface area and dry weight was statistically greater than one, suggesting that as leaf surface area increased, leaf weight increased disproportionately. Moreover, the numerical values of the scaling exponents were affected by habitat type and were correlated with environmental factors, indicating that environmental factors may heavily influence leaf functional traits in this species. Finally, we found significant differences in the AR and SI values across the 10 populations. These differences were linked to distinct habitats. Together, these findings contribute to our comprehension of the adaptive strategies used by *P. subaequalis* under various environmental conditions and provide insights into its ecological evolution.

## FUNDING INFORMATION

This research was supported by Zhejiang Provincial Research Institute Special (2023F1068‐3) (2023) and the Wildlife Plant Protection Management Project of National Forestry Bureau, the Collaborative Innovation Plan of Jiangsu Higher Education, and the Priority Academic Program Development of Jiangsu Higher Education Institutions (PAPD).

## CONFLICT OF INTEREST STATEMENT

All authors declare that they have no conflict of interest.

## Supporting information


Data S1.

Data S2.

## Data Availability

We agree to archive the data associated with this manuscript should the manuscript be accepted.
