# Supplementary material for: Leaf functional traits of Parrotia subaequalis from different environments in eastern China
Source: Plant Environ Interact. 2024 Aug 24;5(4):e70001. doi: 10.1002/pei3.70001 (PMC11344171; doi:10.1002/pei3.70001)
Supplement: Supplementary file 2 — Data S2. [file PEI3-5-e70001-s001.zip › Appendix_(2).docx]

| **TABLE S2** Leaf traits variation range of 10 populations of *P. subaequalis* | | | | | | | |
| --- | --- | --- | --- | --- | --- | --- | --- |
| No. | Population code | Leaf surface area | | Leaf weight | | Leaf length | |
|  |  | Range | Fold | Range | Fold | Range | Fold |
| 1 | AJ | 26.06-3.85 | 6.77 | 0.22-0.02 | 11.00 | 8.40-2.90 | 2.90 |
| 2 | HN | 29.70-2.68 | 11.08 | 0.19-0.01 | 19.00 | 8.50-2.70 | 3.15 |
| 3 | YXI | 24.61-3.22 | 7.64 | 0.16-0.01 | 16.00 | 8.90-2.70 | 3.30 |
| 4 | CH | 26.73-3.14 | 8.51 | 0.18-0.01 | 18.00 | 7.80-2.60 | 3.00 |
| 5 | JD | 23.65-3.68 | 6.43 | 0.12-0.01 | 12.00 | 8.70-2.30 | 3.78 |
| 6 | YX | 40.33-4.50 | 8.96 | 0.24-0.02 | 12.00 | 11.00-3.30 | 3.33 |
| 7 | SC | 34.23-3.88 | 8.82 | 0.17-0.01 | 17.00 | 9.00-2.70 | 3.33 |
| 8 | TC | 44.04-3.41 | 12.91 | 0.25-0.01 | 25.00 | 11.00-3.00 | 3.67 |
| 9 | JZ | 26.84-1.87 | 14.35 | 0.18-0.01 | 18.00 | 7.60-2.00 | 3.80 |
| 10 | YXII | 31.76-2.30 | 13.81 | 0.27-0.01 | 27.00 | 8.20-2.60 | 3.15 |
| 11 | All populations | 44.04-1.87 | 23.55 | 0.27-0.01 | 27.00 | 11.00-2.00 | 5.50 |

| **TABLE S3** ANOVA summary of leaf traits among 10 populations of *P. subaequalis* | | | |
| --- | --- | --- | --- |
| Population | Leaf area | Leaf Weight | Leaf Length |
|  | Mean ± SD | Mean ± SD | Mean ± SD |
| AJ | 12.87±4.80^b^ | 0.10±0.05^a^ | 5.64±1.14^bc^ |
| HN | 11.46±4.87^cd^ | 0.07±0.04^bcd^ | 5.12±1.12^de^ |
| YXI | 10.04±4.06^e^ | 0.06±0.03^de^ | 5.14±1.12^de^ |
| CH | 11.39±4.37^cd^ | 0.08±0.03^bc^ | 5.37±0.98^cd^ |
| JD | 9.63±4.28^e^ | 0.05±0.03^f^ | 4.75±1.11^f^ |
| YX | 14.13±7.10^a^ | 0.08±0.03^b^ | 5.87±1.53^b^ |
| SC | 11.57±5.75^cd^ | 0.06±0.03^e^ | 5.15±1.24^de^ |
| TC | 14.63±7.53^a^ | 0.08±0.05^b^ | 6.32±1.57^a^ |
| JZ | 11.77±4.99^bc^ | 0.07±0.04^cd^ | 5.32±1.19^de^ |
| YXII | 10.35±4.81^de^ | 0.08±0.04^bc^ | 5.03±1.24^e^ |
| *F* | 14.25 | 22.65 | 20.67 |
| *p* | 0.000 | 0.000 | 0.000 |

The same row a, b, c, d, e, f represents the significance of the differences among different populations, using the multiple comparison (LSD) methods, “Bonferroni” correction.

**Appendix**

**
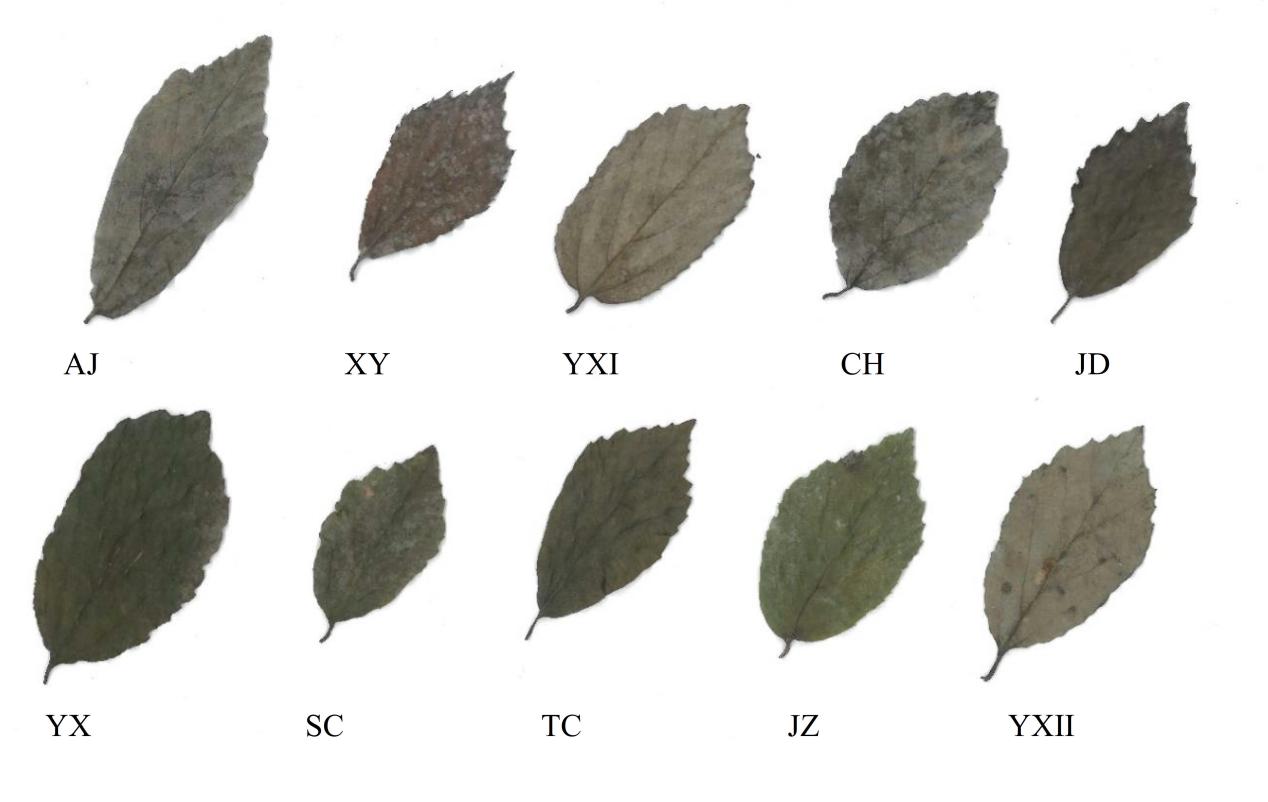
**

**FIGURE S1** Scanned images of typical leaves from each of the 10 populations of *Parrotia subaequalis*.

**FIGURE S2** Examples of scanned and normalized leaves from 10 populations of *Parrotia subaequalis*. (a) Anji; (b) Xinyang; (c) Huangwei; (d) Changhua; (e) Jingde; and (f) Yixing; (g) Shucheng; (h) Tongcheng; (i) Jinzhai; and (j) Hetu. The base for every leaf lies on the coordinate origin. Note the varying axis scales.


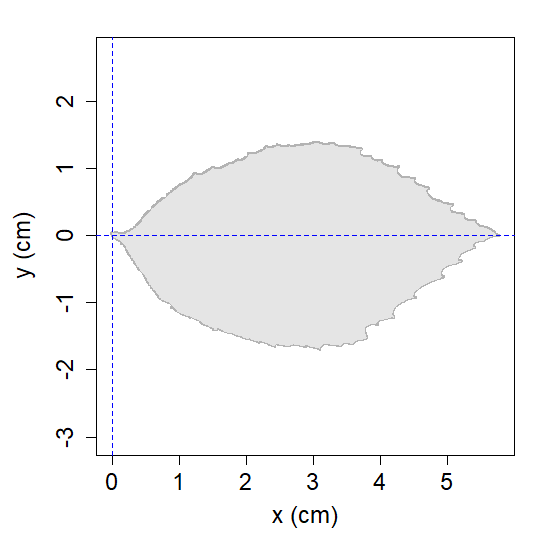

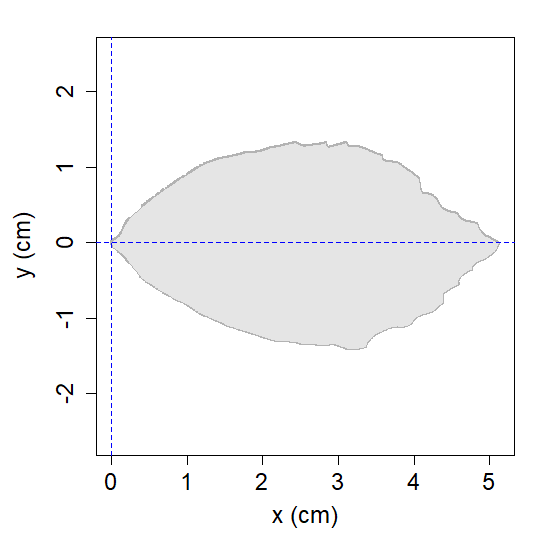

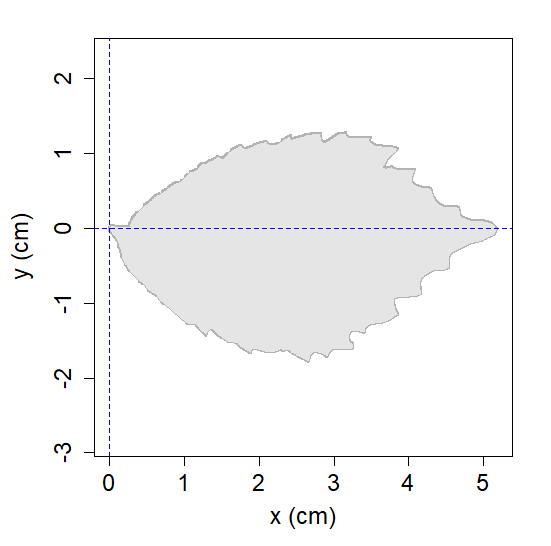

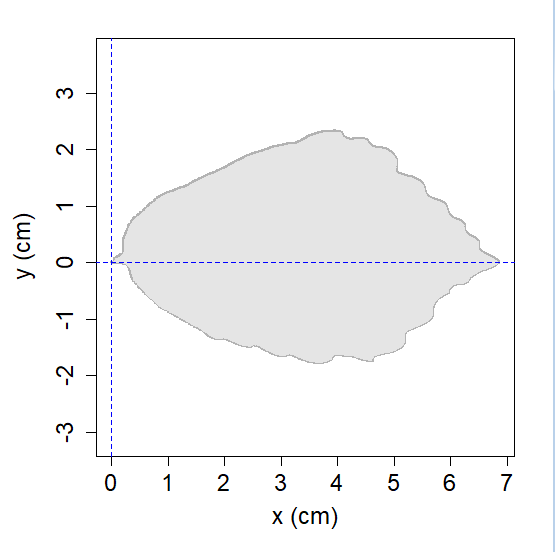

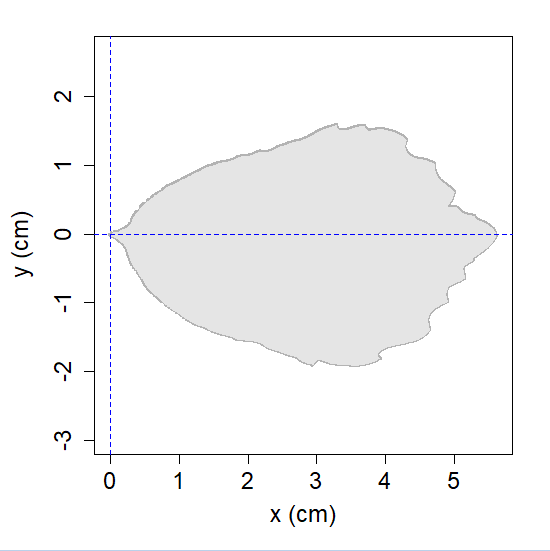

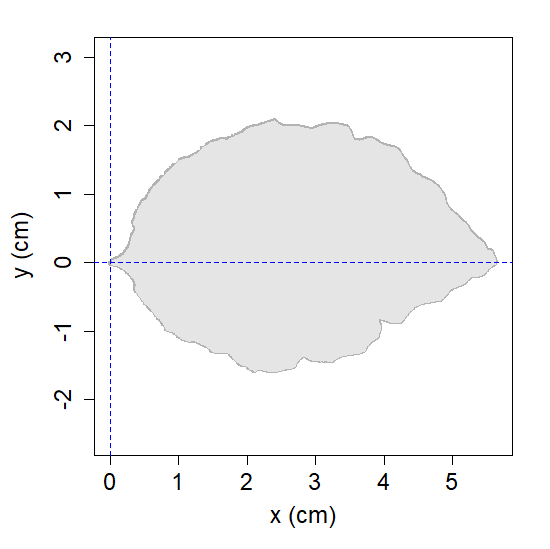

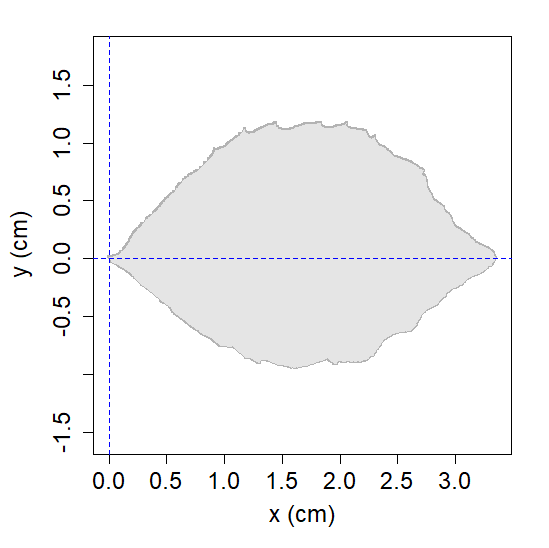

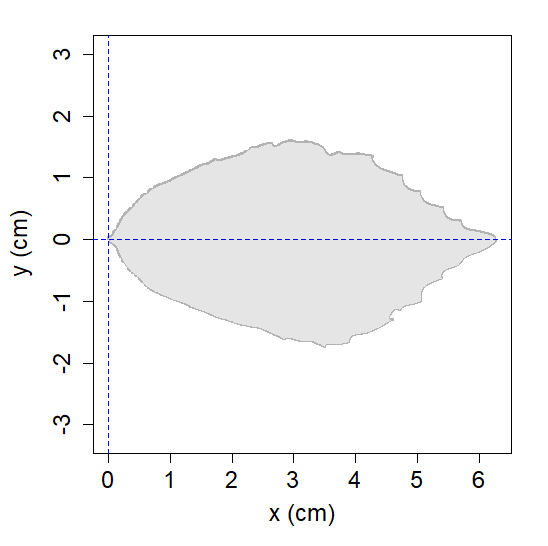

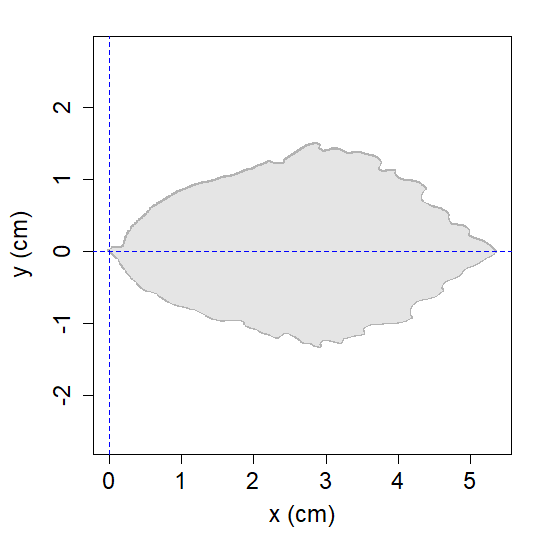

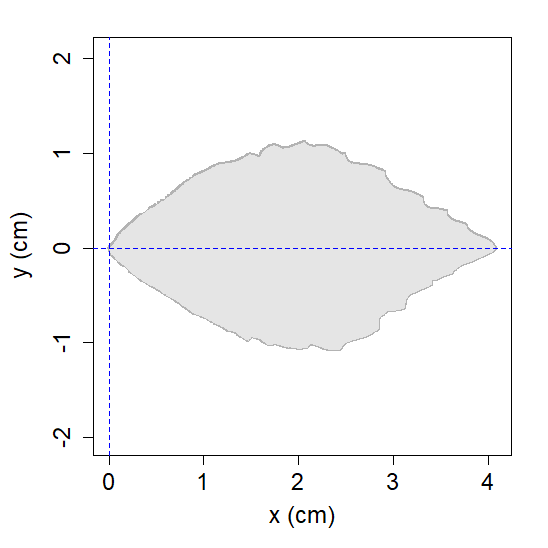


a)

b)

d)

c)

e)

g)

f)

h)

i)

j)


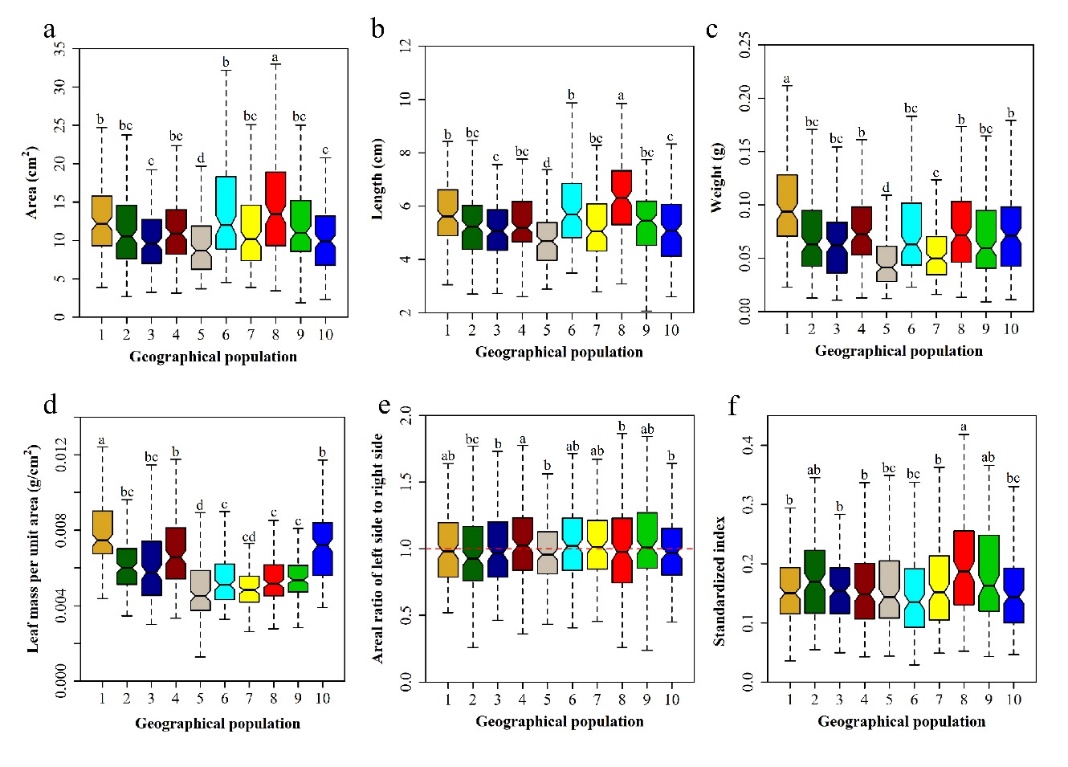


**FIGURE S3** Comparison of mean leaf size, bilateral symmetry, leaf mass per area, and leaf weight values among 10 populations of *Parrotia subaequalis*. In the boxplot, the bold segments in the boxes represent the medians and average level of sampling values and compare the medians to exam the significance of different populations. Means sharing a common capital letter are not significantly different at the alpha = 0.05 level. a, b, c, d represent the significance of the differences. Different colors represent different populations of *P. subaequalis*.


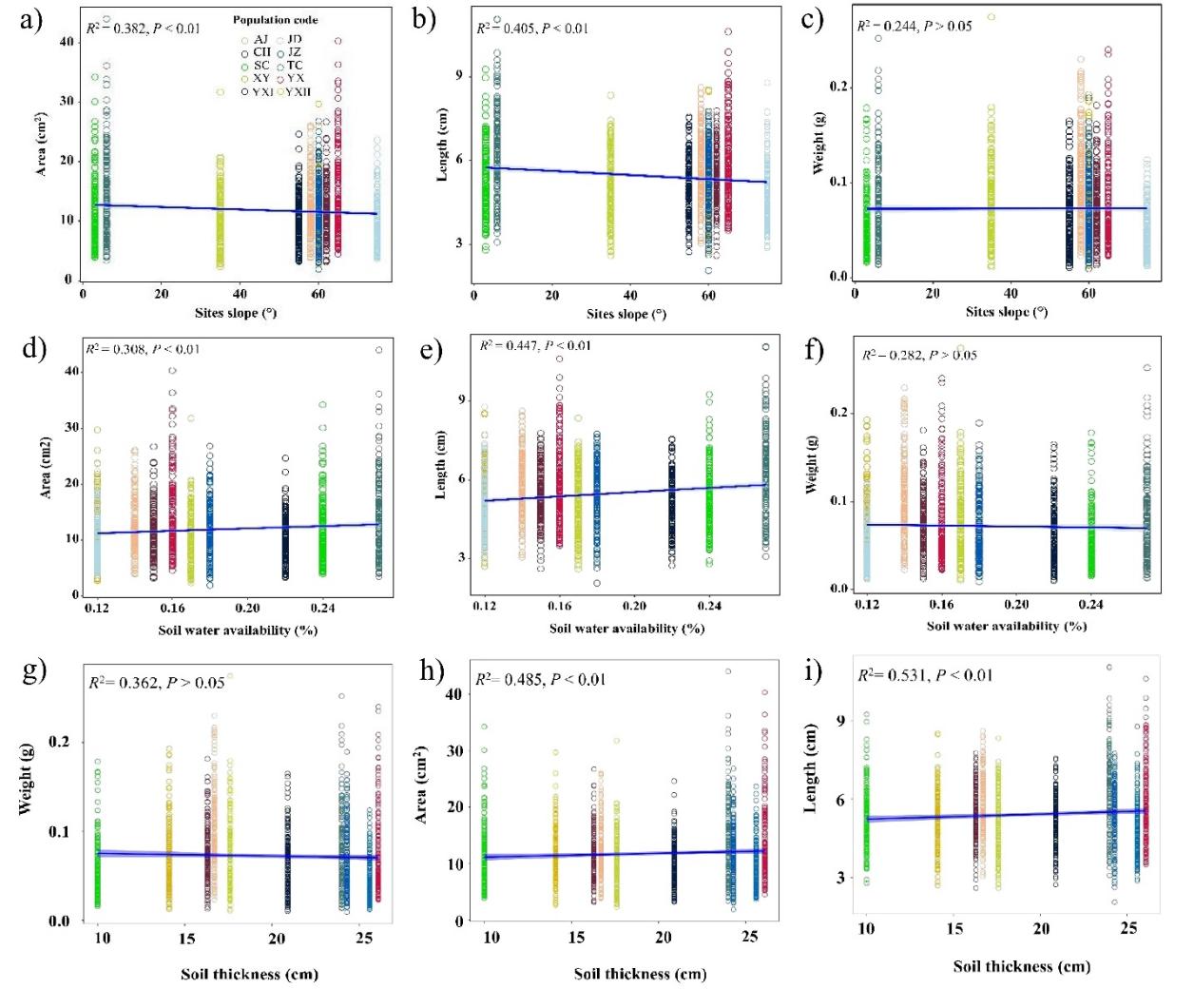


**FIGURE S4** Correlation analysis between leaf area, leaf weight and leaf length and sites slope, soil water availability, and soil thickness. Different colors continuity dot represents a sampling population of *P. subaequalis* in China.


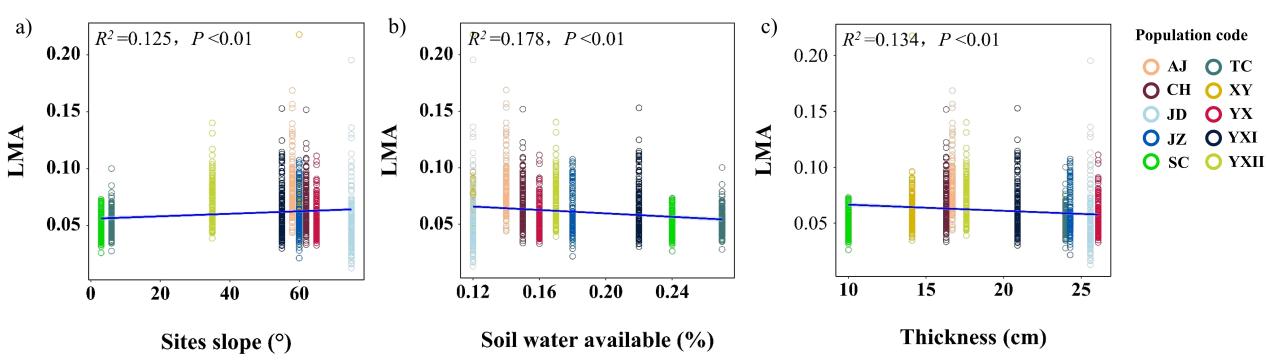


**FIGURE S5** Correlation analysis between LMA and sites slope, soil water availability, and soil thickness. Different colors continuity dot represents a sampling population of *P. subaequalis* in China.

**
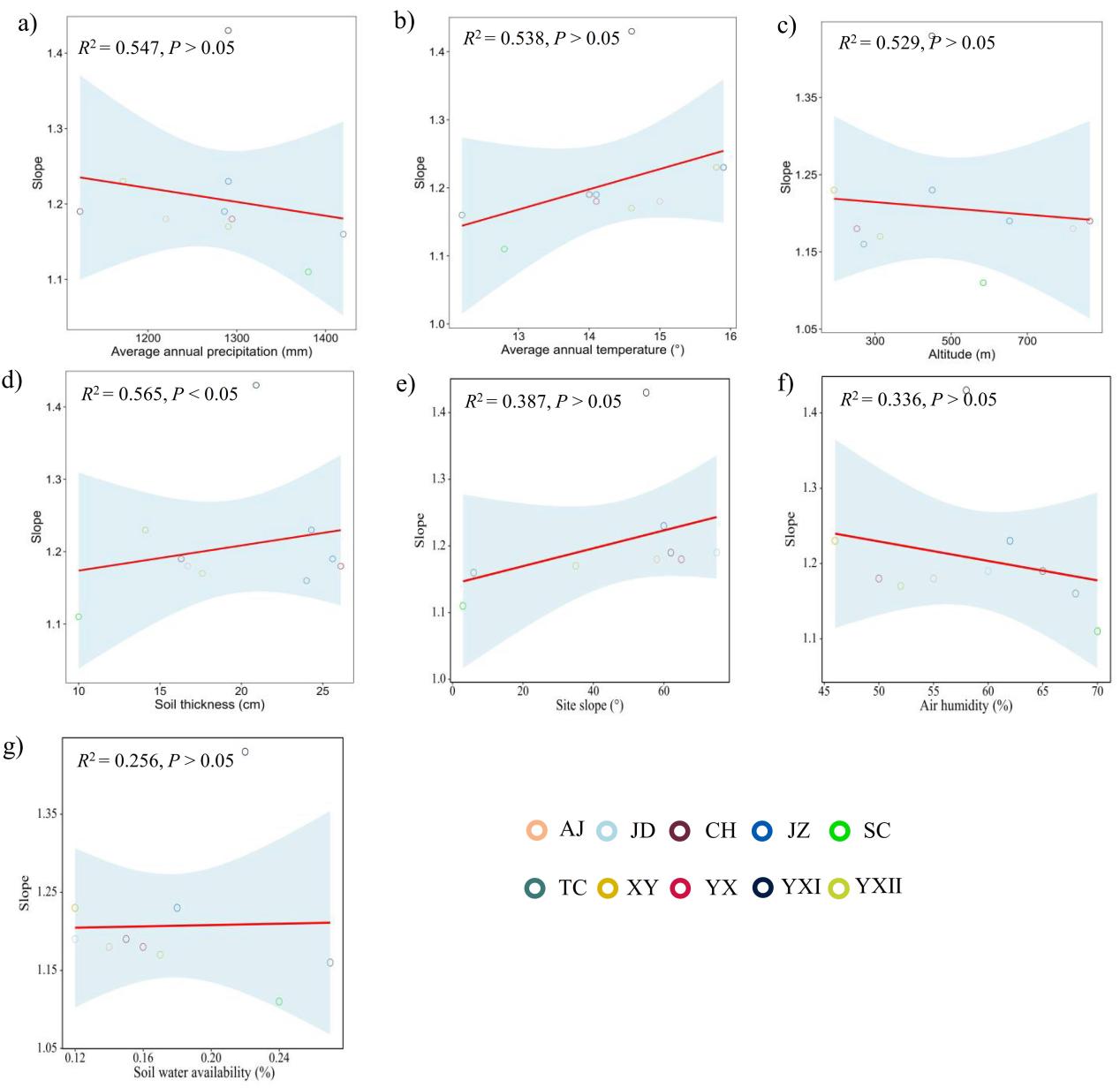
**

**FIGURE S6** Correlation analysis between slope of each population and environmental factors. Different colors represent different populations of *P. subaequalis*.
